# 异基因造血干细胞移植治疗54例骨髓增生异常综合征转化急性髓系白血病的疗效和预后分析

**DOI:** 10.3760/cma.j.cn121090-20231101-00243

**Published:** 2024-04

**Authors:** 书连 陈, 圆圆 施, 利宁 张, 明 龚, 潇予 张, 小利 赵, 梦泽 郝, 嘉璘 魏, 祎 何, 四洲 冯, 明哲 韩, 尔烈 姜

**Affiliations:** 1 中国医学科学院血液病医院（中国医学科学院血液学研究所），血液与健康全国重点实验室，国家血液系统疾病临床医学研究中心，细胞生态海河实验室，天津 300020 State Key Laboratory of Experimental Hematology, National Clinical Research Center for Blood Diseases, Haihe Laboratory of Cell Ecosystem, Institute of Hematology & Blood Diseases Hospital, Chinese Academy of Medical Sciences & Peking Union Medical College, Tianjin 300020, China; 2 天津医学健康研究院，天津 301600 Tianjin Institutes of Health Science, Tianjin 301600, China

**Keywords:** 骨髓增生异常综合征, 白血病转化, 造血干细胞移植, Myelodysplastic syndromes, Leukemia transformation, Hematopoietic stem cell transplantation

## Abstract

**目的:**

评估异基因造血干细胞移植（allo-HSCT）治疗骨髓增生异常综合征转化急性髓系白血病（MDS-AML）的疗效。

**方法:**

收集2018年1月至2022年8月在中国医学科学院血液病医院接受allo-HSCT的MDS-AML患者，对其临床资料进行回顾性分析。

**结果:**

54例患者纳入研究，男26例，女28例，中位年龄46（9～57）岁。53例患者获得粒系造血重建。中位随访597（15～1 934）d。移植后1年总生存（OS）率、无病生存（DFS）率分别为（75.8±5.8）％、（72.1±6.1）％，累积复发率（CIR）为（12.7±4.9）％，非复发死亡率（NRM）为（17.1±5.2）％；移植后3年OS率、DFS率分别为（57.8±7.5）％、（58.1±7.2）％，CIR为（23.2±6.6）％，NRM为（23.7±6.6）％。急性移植物抗宿主病（aGVHD）累积发生率为（57.5±6.9）％，慢性移植物抗宿主病（cGVHD）累积发生率为（48.4±7.7）％。单因素分析显示，移植前造血干细胞移植合并症指数（HCT-CI）评分≥2分、造血植入时骨髓微小残留病（MRD）阳性、发生Ⅲ/Ⅳ度aGVHD、发生细菌/真菌感染以及未发生cGVHD是影响OS的不良因素（*P*<0.05）。多因素因素分析显示，HCT-CI评分≥2分（*P*＝0.001，*HR*＝6.981，95％*CI* 2.186～22.300）、造血植入时骨髓MRD阳性（*P*＝0.010，*HR*＝6.719，95％*CI* 572～28.711）、发生Ⅲ/Ⅳ度aGVHD（*P*＝0.026，*HR*＝3.386，95％*CI* 1.158～9.901）以及cGVHD（*P*＝0.006，*HR*＝0.151，95％*CI* 0.039～0.581）是影响OS的独立危险因素。

**结论:**

对于高复发风险的MDS-AML患者，可考虑早期进行allo-HSCT，移植后加强并发症的处理以及在患者病情允许下尽早开始维持治疗。

## Abstract (en)

**Objective:**

The outcomes of allogeneic hematopoietic stem cell transplantation（allo-HSCT）for myelodysplastic syndromes-evolved acute myeloid leukemia（MDS-AML）were explored.

**Methods:**

A retrospective review was conducted for 54 patients with MDS-AML treated with allo-HSCT in the Institute of Hematology and Blood Disease Hospital from January 2018 to August 2022. The clinical effects after transplantation were observed, and the related risk factors influencing prognosis were explored.

**Results:**

Of the total 54 patients, 26 males, 28 females, and 53 patients achieved hematopoietic reconstruction. After a median follow-up of 597（15–1 934）days, the 1 year overall survival（OS）rate, disease-free survival（DFS）rate, relapse rate（CIR）and non-relapse mortality（NRM）rate were（75.8±5.8）％,（72.1±6.1）％,（12.7±4.9）％, and（17.1±5.2）％, respectively. The 3 year estimated OS, DFS, CIR, and NRM rates were（57.8±7.5）％,（58.1±7.2）％,（23.2±6.6）％, and（23.7±6.6）％, respectively. The cumulative incidence of acute graft-versus-host disease（aGVHD）was（57.5±6.9）％, and the cumulative incidence of chronic graft-versus-host disease（cGVHD）was（48.4±7.7）％. Hematopoietic cell transplantation comorbidity index（HCT-CI）before transplantation was ≥2, minimal residual disease（MRD）was positive on the day of reconstitution, grade Ⅲ/Ⅳ aGVHD, bacterial or fungal infection and no cGVHD after transplantation were adverse prognostic factors for OS（*P*<0.05）. COX regression model for multivariate analysis showed that HCT-CI score before transplantation, bone marrow MRD on the day of response, grade Ⅲ or Ⅳ aGVHD, and cGVHD after transplantation were the independent adverse factors for OS（*P*＝0.001, *HR*＝6.981, 95％*CI* 2.186–22.300; *P*＝0.010, *HR*＝6.719, 95％*CI* 1.572–28.711; *P*＝0.026, *HR*＝3.386, 95％*CI* 1.158–9.901; *P*＝0.006, *HR*＝0.151, 95％*CI* 0.039–0.581）.

**Conclusion:**

For patients with MDS-AML and high risk of relapse, allogeneic transplantation must be considered as soon as possible. The enhanced management of post-transplantation complications and maintenance treatment should be provided whenever possible after transplantation.

骨髓增生异常综合征（MDS）是起源于造血干细胞的异质性髓系肿瘤，临床表现为外周血细胞一系或多系减少，骨髓病态造血及无效造血，高风险向急性髓系白血病（AML）转化[1]–[2]。骨髓增生异常综合征转化急性髓系白血病（MDS-AML）疾病高危、预后差，异基因造血干细胞移植（allo-HSCT）是该病的主要治疗手段。目前对于MDS-AML患者进行allo-HSCT的时机和移植前是否需要化疗尚不明确。我们对近年来在本中心接受allo-HSCT的54例MDS-AML患者进行回顾性分析，观察移植后疗效及影响预后的危险因素，探讨移植的合适时机。

## 病例与方法

1. 病例：2018年1月至2022年8月于我中心接受allo-HSCT治疗的54例MDS-AML患者，其中男26例，女28例，中位年龄46（9～57）岁；采用WHO（2022）标准进行诊断和分型。患者临床特征见表1。

**表1:** 54例MDS-AML患者的临床特征

临床特征	结果
年龄［岁，*M*（范围）］	46（9~57）
性别［例（%）］	
男	26（48.1）
女	28（51.9）
转白前诊断［例（%）］	
MDS-IB1	14（25.9）
MDS-IB2	27（50.0）
其他	13（24.1）
确诊到移植时间［月，*M*（范围）］	4.0（0.5~8.5）
移植前治疗［例（%）］	
是	41（75.9）
否	13（24.1）
移植前骨髓原始细胞［例（%）］	
<5%	23（42.6）
5%~20%	15（27.8）
>20%	16（29.6）
供者类型［例（%）］	
同胞全相合	21（24.8）
单倍体	32（59.3）
无关	1（1.9）
干细胞来源［例（%）］	
外周血+骨髓	3（5.6）
外周血	51（94.4）
MNC回输量［×10^8^/kg，*M*（范围）］	11.68（7.12~24.64）
CD34^+^细胞回输量［×10^6^/kg，*M*（范围）］	3.24（2.00~11.63）
粒细胞植入［例（%）］	53（98.1）
粒细胞植入时间［d，*M*（范围）］	13（10~26）
血小板植入［例（%）］	52（96.3）
血小板植入时间［d，*M*（范围）］	16（8~43）

**注** MDS-AML：骨髓增生异常综合征转化急性髓系白血病；MNC：单个核细胞；MDS-IB1/IB2：骨髓增生异常肿瘤伴原始细胞增多Ⅰ型/Ⅱ型

2. 移植前治疗：54例患者中，13例（24.1％）移植前未治疗。41例（75.9％）接受了化疗或者去甲基化治疗，中位化疗次数2（1～4）次，其中23例治疗后完全缓解（CR），18例治疗后未缓解（NR）。确诊至移植中位间隔时间为4.0（0.5～8.5）个月。

3. 供者及干细胞情况：54例患者中，同胞全相合移植21例，单倍体移植32例，无关供者全相合移植1例。外周血造血干细胞移植51例，骨髓＋外周血造血干细胞移植3例。回输单个核细胞中位数为11.68（7.12～24.64）×10^8^/kg，回输CD34^+^细胞中位数为3.24（2.00～11.63）×10^6^/kg。

4. 预处理方案：所有患者均采用清髓性预处理方案，绝大多数是在经典的白消安+环磷酰胺基础上联合氟达拉滨、阿糖胞苷及5 d地西他滨方案。

5. 移植物抗宿主病（GVHD）防治：同胞全相合移植患者采用环孢素A或他克莫司联合短疗程甲氨蝶呤预防GVHD，单倍体及无关供者移植患者再联合抗胸腺细胞球蛋白和霉酚酸酯以增加免疫抑制强度。

6. 随访和定义：随访截止日期为2023年4月20日。粒细胞植入：连续3 d中性粒细胞计数（ANC）≥0.5×10^9^/L；血小板植入：连续7 d血小板计数≥20×10^9^/L且脱离血小板输注。总生存（OS）时间：从造血干细胞回输开始至患者死亡或末次随访；无病生存（DFS）时间：从造血干细胞回输开始至复发、死亡或末次随访。

7. 统计学处理：应用SPSS23.0软件进行数据分析。OS、DFS应用Kaplan-Meier曲线分析，组间比较应用Log-rank检验，将单因素分析*P*≤0.10的因素纳入Cox回归模型进行多因素分析。*P*<0.05为差异有统计学意义。

## 结果

1. 造血重建：54例MDS-AML患者中53例获得粒细胞植入，中位植入时间为13（10～26）d。52例获得血小板植入，中位植入时间为16（8～43）d。

2. 生存：中位随访时间597（15～1 934）d。至随访截止，54例MDS-AML患者中34例存活，其中33例为无病生存。20例死亡（复发死亡9例，非复发死亡11例）。移植后1年OS率、DFS率、累积复发率（CIR）、非复发死亡率（NRM）分别为（75.8±5.8）％、（72.1±6.1）％、（12.7±4.9）％、（17.1±5.2）％。预期3年OS率、DFS率、CIR、NRM分别为（57.8±7.5）％、（58.1±7.2）％、（23.2±6.6）％、（23.7±6.6）％。生存曲线见图1。

**图1:**
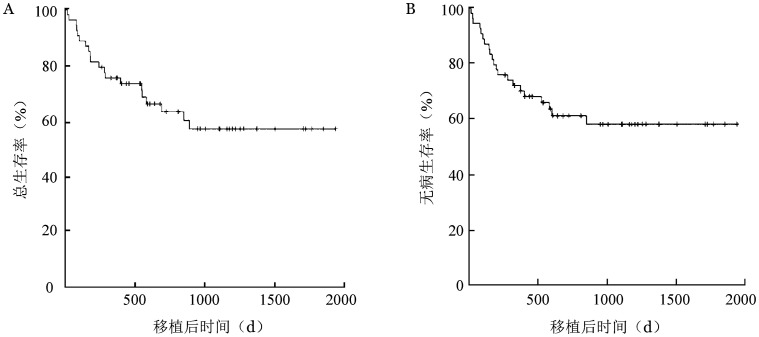
54例骨髓增生异常综合征转化急性髓系白血病（MDS-AML）患者allo-HSCT后总生存曲线（A）和无病生存曲线（B）

3. 移植后并发症：移植后30例患者发生急性GVHD（Ⅰ/Ⅱ度14例，Ⅲ/Ⅳ度16例），中位发生时间为移植后30（13～60）d，急性GVHD累积发生率为（57.5±6.9）％。21例患者发生慢性GVHD（局限型9例，广泛型12例），存活100 d以上患者慢性GVHD累积发生率为（48.4±7.7）％。35例（64.8％）患者发生细菌/真菌感染（肺部感染23例、肠道感染8例、败血症3例、中枢神经系统感染1例），31例（57.4％）患者发生巨细胞病毒（CMV）血症，20例（37.0％）患者发生出血性膀胱炎。

4. 移植后维持治疗：54例MDS-AML患者中21例在移植后接受了维持治疗，中位疗程为3（1～8）个月，其中19例皮下注射阿扎胞苷，1例口服维奈克拉，1例口服索拉非尼。

5. 移植前疾病状态及移植后维持治疗对生存的影响：54例患者如按照移植前治疗情况分为未治疗组（13例）、治疗后完全缓解组（23例）、治疗后未缓解组（18例），移植后3年OS率分别为（45.8±16.7）％、（75.7±9.8）％、（42.9±12.1）％（*P*＝0.09），DFS率分别为（49.2±16.7）％、（72.7±9.6）％、（42.9±12.1）％（*P*＝0.137）；移植后维持治疗组（21例）和未维持治疗组（33例）移植后3年OS率分别为（62.7±17.1）％、（50.2±8.9）％（*P*＝0.058），DFS率分别为（75.0±11.3）％、（48.1±8.8）％（*P*＝0.026）。

6. 预后影响因素的单因素及多因素分析：单因素分析显示患者移植前造血干细胞移植合并症指数（HCT-CI）评分≥2分、植入时骨髓微小残留病（MRD）阳性、移植后发生Ⅲ/Ⅳ度急性GVHD、合并细菌/真菌感染以及未发生慢性GVHD均为OS的不良影响因素（*P*<0.05）；而患者性别、年龄、欧洲白血病网（ELN）分组、移植前治疗及疾病状态、合并CMV血症、维持治疗等对OS均无显著影响（*P*>0.05）（表2）。将移植前HCT-CI评分、移植前治疗、移植前疾病状态、回输当日骨髓MRD、造血植入时骨髓MRD、移植后合并细菌/真菌感染、Ⅲ/Ⅳ度急性GVHD、慢性GVHD及维持治疗纳入多因素分析，结果显示HCT-CI评分（*P*＝0.001，*HR*＝6.981，95％*CI* 2.186～22.300）、植入时骨髓MRD阳性（*P*＝0.010，*HR*＝6.719，95％*CI* 1.572～28.711）、发生Ⅲ/Ⅳ度急性GVHD（*P*＝0.026，*HR*＝3.386，95％*CI* 1.158～9.901）以及慢性GVHD（*P*＝0.006，*HR*＝0.151，95％*CI* 0.039～0.581）是OS的独立危险因素（表3）。

**表2:** 54例MDS-AML患者allo-HSCT后总生存（OS）及无病生存（DFS）影响因素的单因素分析

预后因素	例数	OS		DFS	
		率（%，*x±s*）	*P*值	率（%，*x±s*）	*P*值
患者年龄			0.193		0.052
<50岁	43	62.4±8.4		64.3±7.9	
≥50岁	11	36.4±16.3		34.1±15.0	
供患者性别组合			0.196		0.301
男→男	14	44.4±14.8		46.9±14.1	
男→女	20	52.9±11.6		54.0±11.4	
女→男	12	78.6±13.9		74.1±12.9	
女→女	8	62.5±21.3		64.3±21.0	
ELN分组			0.679		0.707
标危	1	100		100	
中危	13	50.5±14.8		51.3±14.6	
高危	40	59.0±8.9		59.7±8.3	
转白前诊断			0.312		0.303
MDS-IB1	14	38.6±16.0		40.2±15.9	
MDS-IB2	27	57.9±10.5		57.2±10.0	
其他	13	76.2±12.1		76.9±11.7	
移植前治疗及疗效			0.090		0.137
未治疗	13	45.8±16.7		49.2±16.7	
治疗后CR	23	75.7±9.8		72.7±9.6	
治疗后NR	18	42.9±12.1		42.9±12.1	
移植前HCT-CI评分			0.039		0.052
<2分	46	65.1±7.9		64.9±7.6	
≥2分	8	25.0±15.3		25.0±15.3	
供者类型			0.489		0.641
同胞全合	21	53.3±11.8		56.7±10.9	
单倍体	32	60.1±9.7		59.2±9.3	
无关	1	100		100	
回输当日骨髓MRD			0.091		0.160
阴性	32	72.4±8.7		71.3±8.1	
阳性	21	41.2±11.9		43.4±11.8	
造血植入骨髓MRD			0.016		0.024
阴性	45	65.8±8.0		66.6±7.4	
阳性	7	28.6±17.1		28.6±17.1	
GVHD预防			0.368		0.516
CsA	24	51.6±11.9		53.5±11.5	
FK506	30	61.8±9.8		61.6±9.2	
CMV血症			0.185		0.150
无	23	70.4±10.5		73.1±9.4	
有	31	48.1±10.2		47.0±9.9	
细菌/真菌感染			0.006		0.013
无	19	79.1±13.8		82.8±9.1	
有	35	46.0±8.8		46.5±8.8	
Ⅲ/Ⅳ度急性GVHD			0.035		0.042
无	38	65.1±8.9		66.0±8.1	
有	16	41.7±12.9		41.7±12.9	
慢性GVHD			0.006		0.005
无	33	48.0±9.3		47.6±8.8	
有	21	75.9±10.6		76.9±10.2	
移植后维持治疗			0.058		0.026
无	33	50.2±8.9		48.1±8.8	
有	21	62.7±17.1		75.0±11.3	

**注** MDS-AML：骨髓增生异常综合征转化急性髓系白血病；MDS-IB1/IB2：骨髓增生异常肿瘤伴原始细胞增多Ⅰ型/Ⅱ型；ELN：欧洲白血病网；CR：完全缓解；NR：未缓解；HCT-CI：造血干细胞移植合并症指数；MRD：微小残留病；GVHD：移植物抗宿主病；CsA：环孢素A；FK506：他克莫司；CMV：巨细胞病毒

**表3:** 影响54例MDS-AML患者allo-HSCT后总生存及无病生存的多因素分析结果

预后因素	总生存			无病生存		
	*P*值	*HR*	95%*CI*	*P*值	*HR*	95%*CI*
HCT-CI评分（<2分，≥2分）	0.001	6.981	2.186~22.300	0.001	7.298	2.242~23.751
植入时骨髓MRD（阴性，阳性）	0.010	6.719	1.572~28.711	0.017	5.401	1.355~21.523
Ⅲ/Ⅳ度急性GVHD（无，有）	0.026	3.386	1.158~9.901	0.016	3.936	1.295~11.965
慢性GVHD（无，有）	0.006	0.151	0.039~0.581	0.004	0.150	0.041~0.548

**注** MDS-AML：骨髓增生异常综合征转化急性髓系白血病；HCT-CI：造血干细胞移植合并症指数；MRD：微小残留病；GVHD：移植物抗宿主病

## 讨论

高危MDS向AML转化的风险很高，一旦转化为AML后，病程进展快、生存期短，allo-HSCT是有望治愈的唯一手段[3]。有学者建议高危MDS如果移植前骨髓原始细胞≥10％，可以应用去甲基化（HMA）治疗或化疗降低肿瘤负荷后再行移植，可减少移植后复发[4]–[5]。但多数研究者更倾向于直接移植[6]–[10]。Schroeder等[11]回顾性分析了165例MDS和继发性AML患者，67例直接移植，98例移植前接受化疗或HMA治疗，直接移植组、化疗组、HMA组移植后5年OS率分別为61％、50％、45％（*P*＝0.116），DFS率分別为38％、41％、38％（*P*＝0.926），CIR和NRM差异也没有统计学意义，提示移植前治疗与否并不影响移植疗效。郑州大学第一附属医院的一项Meta分析也说明移植前接受HMA与接受支持治疗的MDS患者移植后OS差异无统计学意义，移植前HMA治疗并未使MDS患者的长期生存获益[12]。Chen等[13]回顾性分析了南方医院124例行allo-HSCT的MDS-EB1和MDS-EB2患者，移植组、移植前化疗组、移植前HMA组的移植后5年OS率分别为77.3％、64.3％、68.8％（*P*＝0.047），提示对于高危组MDS患者来说，直接移植组OS优于移植前治疗组。本研究结果显示移植前是否治疗及移植前骨髓原始细胞比例高低（是否>10％或20％）均对患者的预后无明显影响，如果将移植前治疗组进一步细分，CR组和NR组相比，患者的OS显著增高［（75.7±9.8）％对（42.9±12.1）％，*P*＝0.038］，说明移植前治疗获得CR的患者具有较好的生存，但是由于MDS-AML患者化疗缓解率较低、骨髓抑制期长及继发感染风险增加，移植前化疗可能使患者错失移植的最佳时机，所以我们认为对于MDS-AML患者，尽快行allo-HSCT可能是较为合理的选择。

本研究移植后维持治疗组3年DFS率显著优于未维持治疗组［（75.0±11.3）％对（48.1±8.8）％，*P*＝0.026］，两组的OS差异无统计学意义（*P*＝0.058），可能与患者病例数较少相关。多数研究建议患者血象稳定（PLT≥50×10^9^/L）后尽快开始维持治疗，大多从移植后2～3个月开始，持续1年左右。Najima等[14]分析了allo-HSCT后给予阿扎胞苷维持治疗的15例MDS和MDS-AML患者，证实最佳方案为每28天给予阿扎胞苷30 mg/m^2^连续5 d，移植后2年OS率、DFS率分别为77.0％、73.3％。Marini等[15]对移植后用小剂量阿扎胞苷预防及抢先治疗的32例MDS和AML患者进行回顾性分析，发现预防治疗组（每28天阿扎胞苷32 mg/m^2^，连续5 d）移植后1年DFS率高达95％，而抢先治疗组（每28天阿扎胞苷75 mg/m^2^，连续5～7 d）在部分患者联合供者淋巴细胞输注（DLI）情况下，移植后1年DFS率仅为54％，提示在移植后患者身体状况及血象稳定的时候尽早开始维持治疗可以改善患者生存。有研究显示移植后口服阿扎胞苷维持治疗也取得了不错的效果[16]。Wei等[17]在一项前瞻性研究中证实移植后小剂量地西他滨联合维奈克拉维持治疗可降低高危AML和MDS患者的复发率。Oran等[18]进行的一个Ⅲ期随机对照研究纳入187例接受allo-HSCT的AML和MDS患者，发现移植后接受阿扎胞苷维持治疗患者的OS和DFS并没有显著改善。

本组患者的NRM较高，单因素分析提示移植前HCT-CI评分≥2分、移植后合并Ⅲ/Ⅳ度急性GVHD或者感染的患者生存率显著下降。8例HCT-CI评分≥2患者中，3例死于移植后感染、多脏器衰竭等，3例死于复发，2例无病生存，预后明显差于HCT-CI评分0～1的患者，与既往的研究[19]–[21]结论一致。

本研究结果显示移植前是否接受治疗并不影响患者移植后的OS，移植前治疗有反应患者OS较高。移植前HCT-CI评分高、植入时骨髓MRD阳性、移植后发生严重急性GVHD及未发生慢性GVHD是影响生存的独立危险因素，移植后维持治疗可显著改善患者的DFS。对于具有高复发风险的MDS-AML患者，应早期进行allo-HSCT，移植后尽早开始维持治疗。
